# Personal protective equipment stockpile practices: a document and scoping review

**DOI:** 10.1016/j.pmedr.2025.103301

**Published:** 2025-11-04

**Authors:** Caitlin Walker, Eric Toner, Erin Sorrell, Crystal Watson, Tara Kirk Sell

**Affiliations:** a Johns Hopkins Center for Health Security, 700 E. Pratt Street, Suite 900, Baltimore, MD 21202, USA; b Department of Environmental Health and Engineering, Johns Hopkins Bloomberg School of Public Health, 615 N. Wolfe Street, Room E7527, Baltimore, MD 21205, USA

**Keywords:** Personal protective equipment, Strategic stockpile, Stockpile characteristics, Pandemic preparedness, International comparison

## Abstract

**Objective:**

To characterize policies and practices for stockpiling personal protective equipment (PPE) as described in the academic literature, cited documents, and the World Health Organization’s Joint External Evaluation (JEE) and National Action Plan for Health Security (NAPHS) reports, and to identify recommendations for future improvement.

**Methods:**

Seven electronic databases were searched using terms related to PPE and stockpiles: ABI/Inform, CINAHL, EconLit, Embase, Engineering Village, PubMed, and Scopus. Documents published January 2015 to November 2024 were included if substantive detail of a PPE stockpile was described. References were then screened. Available JEE (n = 109) and NAPHS (n = 16) reports were reviewed for stockpile descriptions and recommendations.

**Results:**

The scoping review identified 391 unique records, 13 of which were eligible for inclusion following full-text screening. Seven further documents were identified through reference screening. PPE stockpiles of 19 countries were discussed in included articles, and medical countermeasure stockpiles of 83 countries were discussed in JEE and NAPHS reports. Characteristics and recommendations were identified across all stages of stockpiling.

**Conclusions:**

PPE stockpiles exist in many nations, sharing several characteristics and diverging on others, highlighting potential areas for cross-context learning. Further descriptions and recommendations are needed for stockpiles outside of high-income countries.

## Highlights

• Personal protective equipment stockpiles share both similarities and differences.
• Scoping review documents primarily describe high-income nations' stockpiles.
• World Health Organisation reports identify additional stockpiles.
• Recommendations, primarily US-focused, are made across all stockpile processes.
• Recommendations for a reasonable worst-case pandemic exceed existing targets.

## 1 Introduction

The COVID-19 pandemic demonstrated the inadequacy of personal protective equipment (PPE) stockpiles around the world to meet demands during an emergency (Mehrotra et al., 2020; Davies, 2020; World Health Organization, 2020). Similarly, global manufacturing required months to adequately scale up to meet this need and supply chains showed vulnerability to pandemic-related disruptions (Blueprint Biosecurity, 2024; The Global Fund, 2021). Resulting PPE shortages contributed to lives lost during the pandemic (*Fauci thanks US health workers for sacrifices but admits PPE shortages drove up death toll*, 2021). Countries across many regions faced prolonged shortages, stockpiles of expired items, or shortcomings in PPE distribution (World Health Organization, 2020; Drouard et al., 2023; Cohen and M., 2020; Hoernke et al., 2021; Rajamani et al., 2021). In many nations PPE shortages are widespread even for routine healthcare operations (The Global Fund, 2021). However, levels of PPE shortages were not uniformly distributed amongst nations (The Global Fund, 2021). Elsewhere, such as in Singapore or South Korea, more consistent supplies were sustained throughout the pandemic (Kang et al., 2020; Aloweni et al., 2022). To reduce transmission and lives lost in a pandemic, PPE must be made available as quickly as possible to healthcare workers and the broader workforce required to maintain the functioning of society. Due to manufacturing lead time, supply chain challenges, and limited multinational stockpiles, national-level PPE stockpiles are a key component in achieving this goal (Blueprint Biosecurity, 2024).

In this review the term “personal protective equipment” or “PPE” refers to protective clothing items worn to prevent exposure to biological hazards, including gloves, gowns, masks, and respirators, among other items. The term “stockpile” is used to refer to a reserve of supplies kept for use in a time of emergency need, such as a pandemic. Approaches to PPE stockpiling differ among nations, with some practices and policies likely superior to others. Understanding differing approaches and learning from past experiences can be a valuable starting point to generate recommendations for stockpiles, as has been valuable for US hospital-level stockpiles and international vaccine stockpiles (Rebmann et al., 2017; Yen et al., 2015). For example, lessons learnt from past experiences proved valuable in establishing the World Health Organization’s (WHO’s) oral cholera vaccine stockpile in 2013, guiding decisions made by the technical working group on stockpile use and management, and reducing the time taken for formation (Yen et al., 2015). Similarly, work to understand different national approaches to countermeasure stockpiling in five European countries was commissioned to inform Ireland’s strategy (Health Information and Quality Authority, 2023). The aim of this review is therefore to characterize PPE stockpile policies and practices worldwide as described in academic literature or reports cited in this body of literature, as well as in national reports for WHO Member States. An additional objective is to identify recommendations for future improvement to PPE stockpiles described by these sources.

## 2 Methods

This review included documents from three sources: 1) academic databases, along with cited literature and reports; 2) WHO’s Joint External Evaluation (JEE) Mission Reports; and 3) National Action Plan for Health Security (NAPHS) Reports (Joint External Evaluations, 2024; National Action Plan for Health Security, 2025). National JEE reports provide a standardized voluntary assessment of national compliance under the International Health Regulations (Health Information and Quality Authority, 2023). JEE reports provide high international coverage, serving as a valuable resource regarding PPE stockpiles globally. NAPHS reports describe country-led planning processes towards achieving International Health Regulation capacities, providing detail regarding targets and budget priorities (National Action Plan for Health Security, 2025). These national reports were used to complement a traditional scoping review. This review was based on publicly available documents and therefore exempt from ethical review.

### 2.1 Scoping review

For the scoping review, ABI/Inform, CINAHL, EconLit, Embase, Engineering Village, PubMed, and Scopus were searched for peer-reviewed literature published from 01 January 2015 to 05 November 2024. The methodology adheres to the Preferred Reporting Items for Systematic Reviews and Meta-Analyses guidance for protocol development and scoping reviews (PRISMA-ScR Checklist) (Tricco et al., 2018). Search terms were constructed around two concepts: PPE and stockpiles (see supplementary material I for an example of full search terms).

Duplicate articles were removed, and a reviewer screened each title and abstract for eligibility, followed by full text review. Inclusion criteria required articles to describe substantive details of at least one PPE stockpile. Original studies, reports, commentaries, or reviews were all considered for inclusion, while news reports or webpages were excluded. Exclusion criteria disqualified articles describing PPE stores solely for routine use, or stockpiles of medical countermeasures (MCM) without specific discussion of PPE. References were also checked for inclusion. Online translation services were used to translate any non-English language articles for screening. Data extraction was conducted by the reviewer using a template informed by prior literature, and iteratively updated to include additional categories (Rebmann et al., 2017; Yen et al., 2015; Health Information and Quality Authority, 2023; MedTech Europe, 2020).

### 2.2 JEE and NAPHS reports

All JEE and NAPHS reports available on WHO webpages were downloaded (June-September 2024) and one additional JEE report was retrieved from the Health Security journal (supplementary reference S1) (Joint External Evaluations, 2024; National Action Plan for Health Security, 2025). Relevant report sections were identified and reviewed by the main author (June-October 2024), in addition to key word searches relating to: 1) stockpiles; 2) PPE; and 3) country-specific key terms (see supplementary material II for example search terms). Native speakers were consulted to translate search terms for non-English language documents (French, n = 18; Portuguese, n = 3). Google Translate was then used to translate key sections of the reports prior to review. Data related to medical countermeasure stockpiles was extracted into an Excel-based coding form and new codes were iteratively added as documents were reviewed. Due to inconsistencies in language used to describe stockpiles, nations were categorized as having a stockpile if the term “stockpile” was explicitly used or a store was described for emergency preparedness. All descriptions of MCM stockpiles for biological events were included as some reports did not differentiate MCM from PPE stockpile practices. Descriptions of stockpiles specifically for chemical or radiological events were excluded. Ambiguities arising in inductive coding were resolved through discussion among the authors.

## 3 Results

### 3.1 Attributes of included sources

#### 3.1.1 Scoping review documents

From the scoping review search, 773 records were identified, with 391 remaining after removing duplicates. Seventy-two records remained after title and abstract screening, of which 13 were eligible for inclusion following full-text review (see PRISMA flow diagram in Fig. S1). Seven additional articles were identified through reference review (See Table S1).

The included articles used a range of methodologies to gather information on stockpiles, often combining multiple methods (see list of methodologies in Table S2). The majority were published in 2020 or 2021 and discussed stockpiles at the national level, however several also discussed those at regional (subnational), state, county, and/or hospital levels (see Table S2).

The included articles discussed 19 countries (see Fig. S1), all of which are high-income economies except China, an upper-middle-income economy. All countries discussed were from Europe (n=11), Western Pacific (n=6), or North America (n=2). Seven articles discussed stockpiles of multiple countries. The majority of articles described PPE stockpiles in the US (n = 15), with the next highest number describing stockpiles in the UK (n = 4) (see Fig. S1).

#### 3.1.2 JEE and NAPHS reports

A total of 109 JEE national reports and 16 NAPHS reports were identified and reviewed, all published from 2016 to 2024 (See Table S1). For 83 (76%) nations a MCM stockpile was mentioned, 28 of which described a stockpile specifically of PPE. Fifty-four (65% of those with stockpiles) JEE reports recommended improvements to existing stockpiles, three (19%) NAPHS reports recommended establishing an MCM stockpile, and nine (56%) NAPHS reports included stockpile-specific recommendations in the five-year plan.

The 83 nations with a discussed MCM stockpile include 24 high-income, 17 upper-middle-income, 30 lower-middle-income, and 12 low-income economies. Nations with a discussed MCM stockpile spanned Sub-Saharan Africa (n=27), East Asia and Pacific (n=18), Europe and Central Asia (n=18), Middle East and North Africa (n=12), South Asia (n=5), and North America (n=2).

### 3.2 Stockpile characteristics

Identified policies and characteristics covered multiple stockpile-related processes, including the purpose or mandate, procurement, governance and management, storage and maintenance, allocation, and distribution. WHO reports provided broad global coverage of high-level national strategies (see Table 1). The scoping review identified more nuanced details of PPE stockpile systems for fewer nations (see Table 2).

**Table 1:** Characteristics of medical countermeasure stockpiles, and number of countries for which each characteristic was described in Joint External Evaluation and National Action Plan for Health Security reports published up to September 2024 (total N = 83).

Stockpile Characteristic	N (%)
Conduct of risk or hazard assessments to inform stockpile	10 (12)
Stockpiles at levels other than central (state, regional, and/or territory)	19 (23)
Stockpiles at the hospital or health system level	19 (23)
Hospital or health system stockpiles required	5 (6)
Inventory monitoring and/or management system	8 (10)
Vendor-managed inventory	5 (6)
Existence of a deployment and/or distribution plan	29 (35)
Stockpile simulation or practice exercises conducted	6 (7)

**Table 2:** Personal protective equipment stockpile characteristics and associated locations identified by a scoping review of seven electronic databases including articles published 01 January 2015 to 05 November 2024.

Stockpile Policy or Practice	Location(s)
*Purpose or Mandate*	
PPE^a^ stockpile for pandemic preparedness or public health emergencies	AUS^(S2,S3,S5)^, CAN^(S2,S3,S5)^, CHE^(26)^, CHN^(S3)^, CYP^(S26)^, CZE^(S26)^, DEU^(S25,26)^, FIN^(S26)^, FRA^(S25)^, GBR^(S7–S9,S25)^, ITA^(S26)^, KOR^(S25)^, LUX^(S26)^, MLT^(S26)^, NZL^(S4,6)^, SGP^(S2,S25)^, TWN^(S2,S25)^, USA^(S2,S3,S5,S8,S9,S11–S14,S44,S70,S73–S76)^
Multiple stockpiles of different government departments or agencies	GBR^(S7)^, USA^(S12,S14,S70)^
Developed in preparedness for influenza pandemic	AUS^(S3)^, GBR^(S7–S9)^, NZL^(S6)^
Developed in preparedness for national security threats	AUS^(S3)^, USA^(S11,S12,S73)^
Stockpile of PPE specifically for the healthcare workforce	GBR^(S7)^, NZL^(S4,S6)^, TWN^(S2)^, USA^(S13,S14)^ (partial),
Additional populations covered during the COVID-19 pandemic	NZL^(S4)^, USA^(S13)^
Specified time period for which supplies are intended to last	NZL^(S4)^**,** SGP^(S2)^, TWN^(S2)^, USA^(S9,S11)^ (partial)
*Procurement*	
Centralized procurement process	ESP^(S25)^, GBR^(S7,S8)^, MLT^(S26)^, NZL^(S4)^, TWN^(S2)^
Electronic platform for placing or processing orders	GBR^(S9)^, TWN^(S2)^
Stockpile procurement managed by private contractors	GBR^(S7)^, TWN^(S2)^, USA^(S14)^
*Governance and Management*	
Expert advisory group or process for stockpile	AUS^(S3)^, GBR^(S7)^, USA^(S12)^
Conduct of risk or hazard assessments	AUS^(S3)^, USA^(S12)^
At least one review or audit conducted	AUS^(S5)^, CAN^(S5)^, CZE^(S26)^, NZL^(S4)^, TWN^(S2)^, USA^(S12)^
Stockpile managed by third-party or private contractors	DEU^(S26)^, FIN^(S26)^, GBR^(S7,S9)^, NZL^(S4)^, SGP^(S2,S25)^, TWN^(S2)^, USA^(S12,S14,S44)^
Vendor-managed inventory	NZL^(S4)^, SGP^(S25)^, USA^(S2,S3,S12)^
*Storage and Maintenance*	
Stockpiles at multiple levels (central, state, local, district, and/or city)	AUS^(S3)^, CAN^(S2)^, CHN^(S3)^, DEU^(S26)^, NZL^(S4)^, TWN^(S2)^, USA^(S11–S13,S70,S75,S76)^
Stockpiles at the hospital or health system level	CHE^(S26)^, FRA^(S25)^, TWN^(S2)^, USA^(S70,S75,S76)^
Hospital or health system stockpiles required	CHE^(S26)^, FRA^(S25)^, USA^(S9,S44)^ (partial)
Routine assessment of inventory	TWN^(S2)^, USA^(S14,S70)^ (partial)
Electronic inventory management	USA^(S12,S73,S74)^ (partial)
Controlled storage conditions	TWN^(S2)^, USA^(S12,S70,S75,S76)^ (partial)
Reusable PPE items included in inventory	USA^(S76)^
Shelf-life extension program	AUS^(S5)^, USA^(S5,S12)^
Stock rotation	AUS^(S2,5)^, CHN^(S3)^, NZL^(S4)^ (partial), SGP^(S2)^, TWN^(S2)^
Policies or plans for disposal	USA (partial)^(S70)^
*Allocation*	
Existence of allocation plans or hierarchies	NZL^(S4)^, USA^(S12)^
Criteria previously used for allocation specified	GBR^(S9)^, NZL^(S4)^, TWN^(S25)^, USA^(S11,S13,S74)^
States or local authorities can request items from central stockpile	CAN^(S3)^, CHN^(S3)^, NZL^(S4)^, USA^(S12,S14)^
Regional authorities can request items from neighboring regions	CHN^(S3)^
*Distribution and Dispensing*	
State and local authorities hold responsibility for receipt and distribution	NZL^(S4)^, USA^(S2,S14)^
Use of pharmacies for emergency distribution	KOR^(S25)^, USA^(S12,S13)^
Use of postal service for emergency distribution	TWN^(S25)^
Use of armed forces for emergency distribution	GBR^(S9)^
Third parties contracted for delivery	GBR^(S7,S9)^, NZL^(S4)^, SGP^(S2)^
Practice distribution exercises conducted	USA^(S12)^

S denotes supplementary references.

a Personal protective equipment (PPE)

#### 3.2.1 Purpose or mandate

A purpose of most national PPE stockpiles identified by the scoping review is preparing for public health emergencies or pandemic response (S2,S3). SARS outbreaks were identified as drivers for stockpile development or change in several nations, including New Zealand, Canada, China, and Taiwan (S2-S5). However, PPE stockpiles in Australia, New Zealand, and the UK were specifically designed in preparation for an influenza pandemic (S3,S6,S7-9). In contrast, the South Korean stockpile contains equipment relevant to all eleven of the nation’s priority infectious diseases (S10). While the US Strategic National Stockpile (SNS) was originally developed for response to chemical and biological attacks, its mandate has expanded to cover naturally occurring events, including infectious disease threats (S11,S12).

Several stockpiles specify that PPE items are stockpiled for healthcare workers (S2,S4,S6,S7,S13,S14), however, during the COVID-19 pandemic, some nations expanded this scope to other care providers, essential workers, and the public (S4,S13). Some countries have also set a target duration for which stockpiled PPE should last. For example, in Taiwan’s three-tier system, medical institutions must store 30-days of supplies for epidemic-level use (S2). Goal durations have been discussed of 90 days’ supply for the US SNS, and three to six months’ supply for New Zealand (S4,S11), equivalent to the supply duration held in Singapore (S2). JEE reports for Belgium, Moldova, Qatar, Samoa, Serbia, Seychelles, and Turkmenistan described nations holding three to six months’ worth of MCM supplies (S15-S21). Others described smaller reserves, such as one month in Pakistan and Palau, or 30-days in Tajikistan’s health facilities (S22-S24).

#### 3.2.2 Procurement

Many countries either had an existing centralized procurement process for their PPE stockpile, or established one during the COVID-19 pandemic (S2,S4,S7,S8,S25,S26). Electronic platforms and private contractors have also been used to manage ordering and procurement processes (S2,S7,S9,S14). Some countries incentivized domestic manufacturing of PPE, especially during the COVID-19 pandemic, through investment, subsidies, or guaranteed contracts for stockpile purchases (S2,S4,S7,S8,S11,S25).

#### 3.2.3 Governance and management

The UK, US, and Australia have formal expert advisory groups or processes to inform stockpile contents and practices (S3,S7,S12). Assessments of risk and vulnerability also inform decisions for Australia and the US Veterans Affairs’ stockpiles (S3,S12). 10 nations’ JEE reports also described the conduct of risk-mapping exercises to inform MCM stockpiles (S10,S27-S35).

For many countries, at least one review or audit of the stockpile had been conducted, often by independent bodies (S4-S6,S8,S26). Audits are not always regular, for example 10 years passed without an audit in Canada (S5).

Several examples of multinational coordination were identified, including the European Union’s RescEU stockpile (S8), and Lao’s access to a stockpile of the Association of Southeast Asian Nations in Malaysia (S36). National strategies for several island nations described arrangements with the US SNS to receive supplies including PPE (S23,S37,S38). Division of responsibilities and coordination efforts were also described, such as among government departments in Estonia and Mongolia (S39,S40), and among federal agencies in the US (S12).

External companies contracted for warehousing or stockpile management were described for seven nations in the scoping review. Vendor-managed inventory was discussed in five national JEE reports (S16,S39,S41-S43), and for three countries in the scoping review including the US (S2-S4,S12,S25), where it makes up one of three categories of storage for the SNS (S3).

#### 3.2.4 Storage and maintenance

In many cases stockpiles are held at multiple levels. PPE stockpiles at state, local, district, or city levels were described for seven countries in the scoping review (S3,S6,S9,S44). JEE reports described MCM stockpiles at state, territory, and/or regional levels for 19 countries (S1,S24,S27,S28,S36,S45-S57). MCM stockpiles stored at medical facilities were also noted in 19 JEE reports (S1,S10,S17,S24,S28,S39,S40,S51,S56,S58–S67), with this being a requirement in five countries (S1,S24,S39,S40,S62). New York and California have required hospitals to maintain 90-days of PPE supply, and those in the US Administration for Strategic Preparedness and Response’s Hospital Preparedness Program are required to prepare for a surge in demand (S9,S11,S44). Responsibility for stockpiling lies with healthcare facilities in Switzerland and with employers, including healthcare facilities, in France (S25,S26).

Inventory monitoring or management systems were described in eight JEE reports and several scoping review documents (S10,S28,S45,S51,S56,S60,S68,S69). Many US SNS stockpile sites and the US Veterans Affairs’ stockpile have an annual inventory and an assessment of deficiencies to be addressed (S70). Electronic inventory monitoring systems were identified as a strength in several JEE reports, including for Eswatini, The Gambia, Kenya, Nepal, and South Korea, either at central MCM stores, or to monitor stock across healthcare facilities (S10,S51,S71,S72). Challenges of poor PPE inventory management and data monitoring were noted in several sources for the US, New Zealand, and Canada (S4,S5,S11,S44,S70,S73,S74). Control of warehouse conditions was also mentioned in the scoping review for the US and Taiwan, with a requirement for controlled temperature and humidity in Taiwan and less standardized control of conditions in the US (S2,S75).

Multiple reports described stockpiles holding expired items (S2,S4,S7,S8,S26,S73,S75,S76). Four strategies were identified that could reduce expiration and waste of items: stockpiling reusable PPE; shelf-life extension; returning expired stock; and stock rotation. Storing elastomeric half-mask respirators in the US (less than 1% of respirator models sampled) was the only example of reusable items identified in the review (S76). Shelf-life extension programs were described for the US and Australia, and expired stock may be returned in Australia (S5,S12). Rotation of stockpiled PPE through the hospital system or other entities was described for five countries in the scoping review, ranging widely in extent and expected cost savings. In Australia, only P2 respirators were rotated, and this was estimated to save less than 1% of costs over 10 years (S2,S5). In contrast, the rotation system in Taiwan substantially reduces costs (between one quarter and one half of the cost of each item is paid for surgical masks, N95 respirators, and gowns) (S2). Taiwan’s system requires a minimum PPE stockpile at each of three levels: a central store, local health authorities, and medical facilities, and uses a “first-in-first-out” principle to remove the oldest stock from the central store (S2). Few sources described processes for disposal of expired PPE, however among 15 local hospital stockpiles in the US, eight had written policies for disposing of supplies (S70).

#### 3.2.5 Allocation

Of 19 European countries surveyed, 16 had a system for PPE allocation prioritization, either solely in practice, or in official guidance, usually prioritizing hospital settings (S26). Data used to guide allocation during the COVID-19 pandemic differed slightly among nations but included population, existing supply, rate of use, COVID-19 infections, mortality, hospital capacity, and demographic information (S4,S9,S11,S13,S74). Limited local-level supply and demand data created allocation challenges in the UK and New Zealand, leading to development of an online portal and standardized reporting template (S4,S7,S9). Since the pandemic, US legislation has also been introduced to require greater transparency in PPE allocation (S11). Stockpile allocation was not a focus of JEE or NAPHS reports.

National-level stockpiles often supplement lower-level jurisdictions’ supplies. For example, in the US, New Zealand, and Canada, states or municipalities can request additional supplies in emergencies (S3,S4,S12,S14). Additionally, in China, a region experiencing shortages may request resources from neighboring regions prior to making a request to the central stockpile (S3).

#### 3.2.6 Distribution

Responsibility for distribution lies with different authorities depending on national context. In Australia and New Zealand, agreements are made between central government agencies and states or territories regarding their respective roles (S3,S4). Similarly, during the COVID-19 pandemic federal agencies allocated and distributed PPE from the US SNS, then states and local authorities had responsibilities to receive, hold, and dispense supplies (S11,S12,S14). Plans for distribution and/or deployment of MCM stockpiles were described in 29 JEE reports, however details were limited (S1,S10,S16–S18,S21,S23,S27,S30–S32,S34,S38,S43,S45,S46,S48,S54, S61–S64,S68,S77–S82).

During the COVID-19 pandemic, countries used different methods to distribute PPE supplies, including via pharmacies in both the US and Korea (S12,S13,S25), through nationalization of the postal service in Taiwan (S25), or use of the armed forces in the UK (S9). In New Zealand, a large business supplies company was enlisted to distribute PPE (S4), and the Civil Air Patrol acted as a partner for delivery in Michigan, US, due to geographical challenges (S12).

MCM stockpile simulation or practice exercises were discussed in six JEE reports, including deployment exercises in Australia and testing receipt and distribution of SNS items in the Marshall Islands (S23,S27,S38,S40,S45,S48), as well as described in the scoping review for New York City and Texas (S12).

### 3.3 Stockpile recommendations

Critiques and recommendations spanning many stockpile processes were also identified (see Table 3, Table 4). Recommendations in WHO reports were often broader compared to more nuanced actions suggested in scoping review documents. Although many documents were national in scope, their recommendations are mostly broad and substantially overlap.

**Table 3:** Recommendations for medical countermeasure stockpiles, and number of countries for which each characteristic was described in Joint External Evaluation and National Action Plans for Health Security reports published up to September 2024 (total N = 83).

Stockpile Feature Recommended	N (%)
*Purpose or Mandate*	
Include One Health considerations for stockpile	8 (10)
Base procurement or location of stockpiles on risk/threat profile	9 (11)
*Procurement*	
Develop an inventory list to be purchased for stockpiles	7 (8)
Increase capacity of stockpiles / procure supplies	22 (27)
Increase capacity of stockpiles / procure supplies specifically for personal protective equipment	6 (7)
Create / improve procurement process for stockpile	2 (2)
*Governance and Management*	
Decide on locations to establish stockpiles	4 (5)
Spend to maintain existing infrastructure	2 (2)
*Storage and Maintenance*	
Create or improve management plan or inventory management	18 (22)
Conduct training, supervision, and/or exercises	8 (10)
Map existing stockpiles, inventory, or related infrastructure	12 (14)
Develop new facilities for stockpiling	5 (6)
Establish stockpiles at multiple locations	4 (5)
Digitalize stockpiles or develop or improve IT systems	4 (5)
Develop shelf-life extension program	1 (1)
Actively rotate inventory	1 (1)
*Distribution and Dispensing*	
Create or improve distribution plan or distribution resources for stockpiles	21 (25)

**Table 4:** Personal protective equipment stockpile recommendations potentially applicable to multiple nations identified by a scoping review of seven electronic databases including articles published 01 January 2015 to 05 November 2024.

*Purpose or Mandate*	
**Ο**	Precisely define the stockpile purpose (S11)
**Ο**	Use a risk-based approach (S12)
**Ο**	Ensure that PPE^a^ stockpiles are prepared for a broader range of pandemics than solely influenza (S26)
**Ο**	Use the stockpile to prepare for extreme events that cannot otherwise be adequately dealt with (S12)
**Ο**	Update stockpile scope over time, allow flexibility to address new threats (S12), and regularly reassess assumptions that inform the type and quantities of PPE stocked (S4)
**Ο**	Ensure correct sizes of PPE items are stockpiled for the existing workforce (S13)
**Ο**	Include consideration of children and other vulnerable populations (S12)
**Ο**	Include asymptomatic infections in demand calculations informing the inventory (S4)
**Ο**	Expand the workforce and range of providers who can use PPE from the stockpile (S4)
*Procurement*	
**Ο**	Consider purchasing materials or products produced domestically (S74)
**Ο**	Establish or maintain a centralized procurement process (S73)
**Ο**	Increase stock of reusable PPE (S13)
*Governance and Management*	
**Ο**	Ensure long-term, sustained funding (S70)
**Ο**	Ensure political support at a high level (S73)
**Ο**	Clearly define and allocate responsibilities for all stockpile processes (S3,S4,S11)
**Ο**	Gather input from experts from many sectors to inform stockpile characteristics (S74)
**Ο**	Include stakeholders from multiple levels in decision making (e.g. local, state, and federal) (S12)
**Ο**	Include industry experts in stockpile governance (S73)
**Ο**	Develop incentives for collaboration and inventory sharing among stockpiles at subnational levels (S11)
**Ο**	Share best practices across jurisdictions (S12)
**Ο**	Introduce tax incentives and/or requirements for workplace stockpiles (S13)
*Storage and Maintenance*	
**Ο**	Improve tracking of stockpiled supplies, including their expiration dates (S11)
**Ο**	Use a barcode system to facilitate movement of supplies between stockpiles at different levels (S12)
**Ο**	Assess stockpiled supplies regularly (S4,S70)
**Ο**	Develop standards for warehouse conditions such as temperature and humidity (S13)
**Ο**	Conduct research to understand PPE item performance following storage in varying conditions (S76)
**Ο**	Conduct cost-benefit analysis by region regarding temperature-control of stockpile facilities (S70)
**Ο**	Establish or maintain a system of stock rotation/replacement (S5,S13,S44,S70,S75)
**Ο**	Develop shelf-life extension programs (S5)
**Ο**	Optimize designated shelf-lives and develop shelf-life standards for PPE items (S13)
**Ο**	Replace used, damaged, or expired items in a timely manner (S70)
*Allocation*	
**Ο**	Define strategies for fair and equitable allocation when supplies are limited (S73,S74)
**Ο**	Make allocation methodology and data used to guide allocation publicly available (S11)
**Ο**	Clearly specify who is eligible to receive PPE from the stockpile (S4)
**Ο**	Ensure allocation is based on up-to-date population data (S4)
**Ο**	Use simulation analyses to inform allocation strategies with an aim to reduce transmission (S44)
**Ο**	Improve data and transparency regarding the PPE supply chain and PPE stocked to inform allocation (S4,S7,S74)
**Ο**	Improve data regarding PPE usage, including real-time tracking to inform allocation (S74)
**Ο**	Use barcodes and web portals for tracking items (S12)
**Ο**	Establish an agency with inventory monitoring responsibility (S44)
*Distribution and Dispensing*	
**Ο**	Conduct practice exercises (S4)
**Ο**	Use data from past performance, practice exercises, and modeling to improve distribution and delivery (S12)
**Ο**	Create detailed operational plans for the stockpile system, including for distribution (S4)
**Ο**	Involve third-party logistics companies prior to an emergency event (S12)
**Ο**	Develop agreements among agencies and with logistics companies to ensure faster transport of supplies in an emergency (S3)
**Ο**	Provide guidance and support for last mile distribution (S12)

S denotes supplementary references.

a Personal protective equipment (PPE).

Recommendations regarding the purpose and mandate of stockpiles included to precisely define the purpose (S11), consider a range of scenarios and base decisions on assessment of risk (S12,S26,S29,S33,S39,S40,S64,S83–S87), and reassess and update this purpose over time (S3,S4,S12). Recommendations also encouraged broadening the workforce covered by the stockpile (S4), stocking correctly sized items for this workforce (S13), considering specific populations (S12), and considering One Health aspects (S23,S55,S64,S83,S88–S91).

In terms of procurement, recommendations included increasing the size of existing stockpiles (S17,S18,S29,S33,S34,S39,S46,S48–S50,S55,S61,S64,S78,S79,S81,S83, S88–S90,S92–S97), developing an inventory list to be purchased (S32,S41,S83,S87,S88,S92,S93), increasing stock of reusable PPE items (S13), establishing a centralized procurement process (S73), and purchasing domestic supplies (S74).

Governance and management recommendations included ensuring political support (S73), ensuring long-term funding (S40,S70,S92), clearly defining and allocating stockpile-related responsibilities (S3,S4,S11), including input from a range of experts and stakeholders in decision making (S12,S73,S74), and improving collaboration and learning among various stockpiles (S11,S12).

Several recommendations for storage and maintenance were focused on improving data and tracking of supplies (S11,S12), including better use of electronic systems for inventory management (S55,S92,S98,S99). Recommendations were also made regarding storage under appropriate conditions (S13,S70,S76), and systems to reduce waste and maintain fresh supplies (S13,S76), such as through shelf-life extension (S5,S13,S40), or rotating stock (S5,S13,S44,S70,S75,S100). Other recommendations from the WHO reports included improving inventory management plans (S10,S16–S18,S32,S33,S36,S46,S47,S55,S62,S77,S83,S86,S88,S90,S92,S98,S101–S103), training and supervision (S27,S30,S36,S48,S51,S60,S83,S92), and establishing new stockpile facilities or stockpiling in multiple locations (S33,S51,S69,S83,S84,S89,S93).

Several recommendations were made to improve distribution and dispensing, including creating detailed plans (S1,S4,S12,S16,S17,S22,S32,S36,S47,S50,S51,S60–S62,S78,S83,S86,S94,S101–S105), discussions and agreements between agencies and logistics companies (S3,S12), and using past experiences, practice exercises, and modeling studies to improve plans (S4,S12). Improving allocation with a defined, publicly available strategy was recommended (S4,S11,S73,S74). Allocation strategies were also recommended to use recent population data, and improved inventory data (S4,S7,S74), which could be achieved through use of barcodes and web portals for tracking (S12), and an agency with responsibility for this task (S44).

## 4 Discussion

Stockpiles described in the scoping review were primarily those of high-income economies in Europe, the Western Pacific, and North America, with a particular focus on US stockpiles. Many more PPE and broader MCM stockpiles were described in JEE and NAPHS reports, spanning countries of all income levels and additional geographic regions. The multitude of US-focused reports demonstrates the complex nature of a stockpile system and provides some broad recommendations; however, this also highlights the existing gap for other nations and the potential benefit of equivalent thorough evaluations. Furthermore, while some identified characteristics were shared among multiple nations, others differed, creating opportunities for knowledge sharing among contexts. The varying strategies employed to minimize waste or expiry of items exemplify such differences, however, discussion of their pros, cons, and barriers to implementation were largely absent, and estimated cost savings, where available, differed greatly between nations. Further research to characterize PPE stockpiles outside of high-income economies would be valuable, in addition to further evaluations of strengths, weaknesses, and cost-effectiveness of stockpile strategies across countries of all income levels.

Various factors likely contribute to stockpile differences, including past outbreaks or events, which have commonly been the impetus for introduction of, or improvements to, national PPE stockpiles, feeding into differing priorities and mandates. Planning beyond preparedness for historical events and using a risk-based approach to inform decisions were recommended for several nations, as well as broadening the event types and workforce accounted for. Other factors resulting in stockpile differences likely include domestic PPE manufacturing capacity, regional agreements, workforce size, political system, and income level. Given such differences, caution is required in generalizing recommendations across contexts, however, many identified recommendations point to broad factors for consideration across various settings.

Political support and ongoing financing were identified as standalone recommendations, and these also underpin many recommended policies and practices. While strong governance and financial support are foundational, other recommendations rely more on improved coordination, management practices, or technological solutions. Examples of areas that may especially benefit from technological improvements include demand forecasting, end-to-end supply chain monitoring, and availability of real-time data to inform allocation. Technological solutions identified included radio-frequency identification tags or two-dimensional barcodes, which can both improve inventory monitoring accuracy and reduce time burdens (S12).

Existing research and policy documents provide largely complementary findings and recommendations. For example, recommendations identified in our review overlapped with future directions suggested for MCM stockpiles in several European countries, which included gathering high-level political support and sufficient budgets, rotating stocks, and maintaining multiple decentralized stockpiles (Health Information and Quality Authority, 2023). Complimentary to our review which identifies recommendations to refine specific existing stockpiles, one report takes a country-agnostic approach to identifying recommendations for a specific feasible worst-case pandemic scenario, using parametric modeling, and gathering solutions from stakeholders (Blueprint Biosecurity, 2024). Some recommendations generated in this report aligned with findings from our review, including the need for long-term funding commitments; rotation of stocks; and increasing data availability and transparency regarding existing stockpiles, supply chains, and demand for PPE (Blueprint Biosecurity, 2024). Recommendations from this report also included stockpiling reusable respirators due to the greater protection, fit, and usability afforded, as well as higher cost-effectiveness over their lifecycle, especially due to reduced stockpile space occupied (Blueprint Biosecurity, 2024). Aside from a small stockpile of elastomeric half-mask respirators in the US, no examples of reusable respirator stockpiling were identified in our review. Based on data regarding manufacturing lead time during the COVID-19 pandemic, the report also concluded that nations should stockpile 150-days’ worth of supply for epidemic-level PPE use (Blueprint Biosecurity, 2024). Several documents in our review did identify estimates of the time period for which stockpiled supplies should last, however, these were frequently much less than 150 days and based on routine use rates. Additional recommendations made by the Blueprint Biosecurity report and not identified in our review included stockpiling of precursor materials and appropriately balancing central stockpiles with user-managed inventory, distributor-managed inventory, and vendor-managed inventory to varying degrees for each PPE item (Blueprint Biosecurity, 2024). Findings from our review therefore underscore the significant gap between current PPE stockpile characteristics and recommendations compared to target requirements to prepare for a realistic worst-case pandemic.

This review has several limitations. Public JEE and NAPHS reports are not available for all nations and were often published prior to the COVID-19 pandemic, failing to capture changes to policies or practices during this event. Despite searching multiple sources, identified literature was also limited and may not include all nations with existing PPE stockpiles. It is further not required for JEE reports to describe PPE stockpiles and practices, and it was not always possible to deduce which characteristics and recommendations pertain to PPE specifically, preventing this distinction from being made in some findings of this review. Despite a clear and consistent definition being applied to identify existing stockpiles, misclassification is possible due to inconsistencies in the language used to describe stockpiles in WHO reports and wide variation in stockpile capacity among nations. While documents were not excluded based on language, use of English search terms could have introduced bias in identified literature and there is potential for translation inaccuracies to have occurred.

## 5 Conclusion

PPE stockpiles are a critical element of pandemic preparedness, serving to provide immediate access to PPE in an emergency event. Many nations globally are implementing some form of PPE stockpile strategy. However, few stockpiles have been characterized in detail or received recommendations in the academic literature. Our review found several shared characteristics among PPE stockpiles, spanning all stages from procurement through to distribution and dispensing, as well as multiple areas in which strategies diverged. While numerous recommendations have been made to improve existing PPE stockpiles, many of these are centered on stockpiles of high-income economies, especially the US, and these fall short of estimated requirements to prepare for a reasonable worst-case pandemic event. Further research is needed to examine the estimated costs and cost-savings of identified recommendations, and their applicability across contexts.

## CRediT authorship contribution statement

**Caitlin Walker:** Writing – review & editing, Writing – original draft, Methodology, Formal analysis, Data curation, Conceptualization. **Eric Toner:** Writing – review & editing, Methodology, Conceptualization. **Erin Sorrell:** Writing – review & editing, Methodology, Conceptualization. **Crystal Watson:** Writing – review & editing, Methodology, Conceptualization. **Tara Kirk Sell:** Writing – review & editing, Supervision, Methodology, Conceptualization.

## Funding

Caitlin Walker is supported by Open Philanthropy and the Bloomberg School of Public Health Lipitz Public Health Award. This research was conducted as part of her dissertation work.

## Declaration of competing interest

The authors declare that they have no known competing financial interests or personal relationships that could have appeared to influence the work reported in this paper.

## Data Availability

N/A. Publicly available data.

## Appendix A Supplementary data

Supplementary material Image 1
