# Supplementary material for: Personal protective equipment stockpile practices: a document and scoping review
Source: Prev Med Rep. 2025 Nov 4;60:103301. doi: 10.1016/j.pmedr.2025.103301 (PMC12744811; doi:10.1016/j.pmedr.2025.103301)
Supplement: Supplementary file 1 — Supplementary material [file mmc1.docx]

Supplementary Material for: Personal Protective Equipment Stockpile Practices: A Document and Scoping Review

**Supplementary material I**

*Scoping Review Search Strategy*

Search Terms Example (PubMed)

("personal protective equipment"[MeSH Terms] OR "Respiratory Protective Devices"[MeSH Terms] OR "personal protective equipment"[tw] OR "glove"[tw] OR "gloves"[tw] OR "gown"[tw] OR "gowns"[tw] OR "respiratory protection"[tw] OR "n95"[tw] OR "respirator"[tw] OR "respirators"[tw] OR "ffr"[tw] OR “personnel protective equipment”[tw] OR "face shield*"[tw] OR "surgical mask*"[tw] OR "face mask*"[tw] OR "goggles"[tw] OR "coverall*"[tw] OR "PAPR"[tw] OR "PAPRs"[tw] OR "SCBA"[tw] OR "SCBAs"[tw] OR "EHMR"[tw] OR "EHMRs"[tw] OR "n99"[tw] OR "n100"[tw] OR "r95"[tw] OR "r99"[tw] OR "r100"[tw] OR "p95"[tw] OR "p99"[tw] OR "p100"[tw] OR “High Efficacy Particulate Air”[tw])

AND

("strategic stockpile"[MeSH Terms] OR "stockpil*"[tw] OR "cache*"[tw] OR "surge capacity"[tw] OR “resilient suppl*”[tw] OR "surge demand"[tw] OR “stock”[tw])

Search Strategy Details

Preliminary searches of PubMed, Embase, Scopus, Cumulative Index to Nursing and Allied Health Literature (CINAHL), and OSF registries were conducted, and no equivalent current or ongoing systematic or scoping reviews were identified.

Included publications were limited to those published since 2015. Stockpile descriptions in literature published greater than 10 years prior were considered likely to be outdated. Database searches were conducted on 05 November 2024. English language search terms were used, however articles were not excluded based on language.

**Supplementary material II**

*Joint External Evaluation and National Action Plan for Health Security Reports Search Strategy*

English Language Search Terms

“stock”, “reserve”, “stor”, “personal protect”, and “PPE”

French Language Search Terms

“stock”, “reserve”, “entrop”, “dépôt”, “protection personnelle”, “de protection”, and “EPI”

Portuguese Language Search Terms

“estoque”, “estocagem”, “reserva”, “provisão”, “abastecimento”, “inventário”, “disponibilização”, “conservação”, “manutenção continua”, “manutenção e gestão”, “equipamento de Protecção”, “equipamento de proteção”, and “EPI”

**Supplementary material III**

**Figure S1.** PRISMA flow diagram of search results for scoping review including articles published 01 January 2015 to 05 November 2024.

Electronic Database searches: PubMed, Embase, Scopus, Engineering Village, EconLit, ABI/Inform, and CINAHL

(n = 773 records)

Records after duplicates were removed

(n = 391)

Titles/abstracts screened

(n = 391)

Full-text articles screened

(n = 72)

Excluded

(n = 319)

Excluded

(n = 59)

Articles discussing characteristics of a PPE stockpile

(n = 13)

+

Articles identified through citation screening

(n = 7)

Countries discussed (number of records): USA (15); UK (4); Canada (3); Singapore (3); Australia (2); France (2); New Zealand (2); Taiwan (2); China (1); Cyprus (1); Czechia (1); Finland (1); Germany (1); Italy (1); Latvia (1); Luxembourg (1); Malta (1); South Korea (1); Switzerland (1)

**Supplementary Material IV**

**Table S1.** Full list of Joint External Evaluation (N=109) and National Action Plan for Health Security reports reviewed (N=16), as well as articles identified by a scoping review of seven electronic databases (N=20). Reports published up to September 2024 were included and the scoping review covers articles published 01 January 2015 to 05 November 2024.

| **Supplementary**  **Reference Number** | **Type** | **Full Reference** |
| --- | --- | --- |
| 1 | JEE | Toner ES, Nuzzo JB, Shearer M, Watson C, Sell TK, Cicero A. The Joint External Evaluation of Taiwan: The External Evaluators’ Perspective. *Health Secur*. 2017;15(2):127-131. doi:10.1089/hs.2016.0109 |
| 2 | Scoping review | Chen YJ, Chiang PJ, Cheng YH, et al. Stockpile Model of Personal Protective Equipment in Taiwan. *Health Secur*. 2017;15(2):170-174. doi:10.1089/hs.2016.0103 |
| 3 | Scoping review | Wang X, Zhang X, He J. Challenges to the system of reserve medical supplies for public health emergencies: reflections on the outbreak of the severe acute respiratory syndrome coronavirus 2 (SARS-CoV-2) epidemic in China. *Biosci Trends*. 2020;14(1):3-8. doi:10.5582/bst.2020.01043 |
| 4 | Scoping review | Controller and Auditor-General. *Ministry of Health: Management of Personal Protective Equipment in Response to Covid-19.*; 2020. Accessed January 30, 2025. https://oag.parliament.nz/2020/ppe/overview.htm |
| 5 | Scoping review | Laing S, Westervelt E. Canada’s National Emergency Stockpile System: time for a new long-term strategy. *CMAJ Can Med Assoc J*. 2020;192(28):E810-E811. doi:10.1503/cmaj.200946 |
| 6 | Scoping review | Fenton E. Management of personal protective equipment in New Zealand during the COVID-19 pandemic: report from the Auditor-General. *N Z Med J*. 2020;133(1522):144-148. |
| 7 | Scoping review | Gareth Davies. *The Supply of Personal Protective Equipment (PPE) during the COVID-19 Pandemic - National Audit Office (NAO) Report*. National Audit Office, Department of Health and Social Care; 2020. Accessed April 7, 2024. https://www.nao.org.uk/reports/supplying-the-nhs-and-adult-social-care-sector-with-personal-protective-equipment-ppe/ |
| 8 | Scoping review | Feinmann J. What happened to our national emergency stockpiles? *BMJ*. 2021;375:n2849. doi:10.1136/bmj.n2849 |
| 9 | Scoping review | Barber A, Vinzent A, Williams I. Management of personal protective equipment during the COVID-19 pandemic in England and the state of New York: A comparative case study. *J Emerg Manag*. 2020;18(7):71-89. doi:10.5055/jem.0531 |
| 10 | *JEE* | *Joint External Evaluation of IHR Core Capacities of the Republic of Korea.* World Health Organisation; 2018. Accessed June 13, 2024. https://www.who.int/publications/i/item/WHO-WHE-CPI-2017.65 |
| 11 | Scoping review | Karen M. Sutter, Michael H. Cecire, Agata Bodie, et al. *COVID-19 and Domestic PPE Production and Distribution: Issues and Policy Options*. Congressional Research Service; 2020. Accessed January 21, 2025. https://crsreports.congress.gov/product/details?prodcode=R46628 |
| 12 | Scoping review | Board on Health Sciences Policy, Health and Medicine Division, National Academies of Sciences, Engineering, and Medicine. *The Nation’s Medical Countermeasure Stockpile: Opportunities to Improve the Efficiency, Effectiveness, and Sustainability of the CDC Strategic National Stockpile: Workshop Summary*. National Academies Press (US); 2016. Accessed January 27, 2025. http://www.ncbi.nlm.nih.gov/books/NBK396382/ |
| 13 | Scoping review | Babik KR, Downey A, Alper J, eds. *Personal Protective Equipment and Personal Protective Technology Product Standardization for a Resilient Public Health Supply Chain: Proceedings of a Workshop*. National Academies Press; 2023. doi:10.17226/27094 |
| 14 | Scoping review | Herrera GJ, Gottron F. National Stockpiles: Background and Issues for Congress. Published online June 15, 2020. https://crsreports.congress.gov/product/pdf/IF/IF11574 |
| 15 | *JEE* | *Joint External Evaluation of IHR Core Capacities of the Republic of Seychelles*. World Health Organisation; 2018. Accessed June 13, 2024. https://www.who.int/publications/i/item/WHO-WHE-CPI-REP-2018.21 |
| 16 | *JEE* | *Joint External Evaluation of IHR Core Capacities of the Kingdom of Belgium.* World Health Organisation; 2017. Accessed June 13, 2024. https://www.who.int/publications/i/item/WHO-WHE-CPI-REP-2017.37 |
| 17 | *JEE* | *Joint External Evaluation of IHR Core Capacities of the Republic of Moldova.* World Health Organisation; 2019. Accessed June 13, 2024. https://www.who.int/publications/i/item/WHO-WHE-CPI-2019.54 |
| 18 | *JEE* | *Joint External Evaluation of IHR Core Capacities of the State of Qatar.* World Health Organisation; 2017. Accessed June 13, 2024. https://www.who.int/publications/i/item/WHO-WHE-CPI-2017.6 |
| 19 | *JEE* | *Joint External Evaluation of IHR Core Capacities of Samoa.* World Health Organisation; 2024. Accessed June 13, 2024. https://www.who.int/publications/i/item/9789240093560 |
| 20 | *JEE* | *Joint External Evaluation of IHR Core Capacities of the Republic of Serbia.* World Health Organisation; 2019. Accessed June 13, 2024. https://www.who.int/publications/i/item/WHO-WHE-CPI-2019.36 |
| 21 | *JEE* | *Joint External Evaluation of IHR Core Capacities of Turkmenistan.* World Health Organisation; 2017. Accessed June 13, 2024. https://www.who.int/publications/i/item/WHO-WHE-CPI-2017.29 |
| 22 | *JEE* | *Joint External Evaluation of IHR Core Capacities of the Islamic Republic of Pakistan.* World Health Organisation; 2017. Accessed June 13, 2024. https://www.who.int/publications/i/item/WHO-WHE-CPI-2017.9 |
| 23 | *JEE* | *Joint External Evaluation of IHR Core Capacities of the Republic of Palau.* World Health Organisation; 2020. Accessed June 13, 2024. https://www.who.int/publications/i/item/9789240008144 |
| 24 | *JEE* | *Joint External Evaluation of IHR Core Capacities of the Republic of Tajikistan.* World Health Organisation; 2021. Accessed June 13, 2024. https://www.who.int/publications/i/item/9789240018242 |
| 25 | *Scoping review* | *International Benchmark Survey of COVID-19 Crisis Management*. EY; 2020. Accessed November 20, 2024. https://assets. ey.com/content/dam/ey-sites/ey-com/fr_fr/topics/health/ey-international-benchmark-crisismanagement- en.pdf |
| 26 | Scoping review | Kroneman M, Williams GA, Winkelmann J, Spreeuwenberg P, Davidovics K, Groenewegen PP. Personal protective equipment for healthcare workers during COVID-19: Developing and applying a questionnaire and assessing associations between infection rates and shortages across 19 countries. *Health Policy Amst Neth*. 2024;146:105097. doi:10.1016/j.healthpol.2024.105097 |
| 27 | *JEE* | *Joint External Evaluation of IHR Core Capacities of Canada.* World Health Organisation; 2019. Accessed June 13, 2024. https://www.who.int/publications-detail-redirect/WHO-WHE-CPI-2019.62 |
| 28 | *JEE* | *Joint External Evaluation of IHR Core Capacities of the Republic of Finland.* World Health Organisation; 2017. Accessed June 13, 2024. https://www.who.int/publications/i/item/WHO-WHE-CPI-2017.24 |
| 29 | *JEE* | *Joint External Evaluation of IHR Core Capacities of the Republic of Ghana.* World Health Organisation; 2017. Accessed June 13, 2024. https://www.who.int/publications/i/item/WHO-WHE-CPI-2017.26 |
| 30 | *JEE* | *Joint External Evaluation of IHR Core Capacities of the Republic of Lithuania.* World Health Organisation; 2019. Accessed June 13, 2024. https://www.who.int/publications-detail-redirect/WHO-WHE-CPI-2019.35 |
| 31 | *JEE* | *Joint External Evaluation of IHR Core Capacities of Malaysia.* World Health Organisation; 2020. Accessed June 13, 2024. https://www.who.int/publications/i/item/9789240015296 |
| 32 | *JEE* | *Joint External Evaluation of IHR Core Capacities of the Kingdom of Morocco.* World Health Organisation; 2017. Accessed June 13, 2024. https://www.who.int/publications/i/item/WHO-WHE-CPI-2017.3 |
| 33 | *JEE* | *Joint External Evaluation of IHR Core Capacities of Nigeria.* World Health Organisation; 2017. Accessed June 13, 2024. https://www.who.int/publications/i/item/WHO-WHE-CPI-REP-2017.46 |
| 34 | *JEE* | *Joint External Evaluation of IHR Core Capacities of the Sultanate of Oman.* World Health Organisation; 2017. Accessed June 13, 2024. https://www.who.int/publications/i/item/WHO-WHE-CPI-REP-2017.59 |
| 35 | *JEE* | *Joint External Evaluation of IHR Core Capacities of Singapore.* World Health Organisation; 2018. Accessed June 13, 2024. https://www.who.int/publications/i/item/WHO-WHE-CPI-REP-2018.25 |
| 36 | *JEE* | *Joint External Evaluation of IHR Core Capacities of the Lao People’s Democratic Republic.* World Health Organisation; 2017. Accessed June 13, 2024. https://www.who.int/publications/i/item/WHO-WHE-CPI-REP-2017.35 |
| 37 | *JEE* | *Joint External Evaluation of IHR Core Capacities of the Federated States of Micronesia.* World Health Organisation; 2019. Accessed June 13, 2024. https://www.who.int/publications/i/item/WHO-WHE-CPI-2018.34 |
| 38 | *JEE* | *Joint External Evaluation of IHR Core Capacities of the Republic of the Marshall Islands.* World Health Organisation; 2020. Accessed June 13, 2024. https://www.who.int/publications/i/item/9789240008168 |
| 39 | *JEE* | *Joint External Evaluation of the International Health Regulations (‎2005)‎ Core Capacities for Estonia: Mission Report, 9-13 October 2023*. World Health Organisation; 2024. Accessed June 13, 2024. https://www.who.int/publications/i/item/9789240092921 |
| 40 | *JEE* | *Joint External Evaluation of IHR Core Capacities of Mongolia.* World Health Organisation; 2017. Accessed June 13, 2024. https://www.who.int/publications/i/item/WHO-WHE-CPI-REP-2017.51 |
| 41 | *JEE* | *Joint External Evaluation of IHR Core Capacities of the Swiss Confederation and the Principality of Liechtenstein.* World Health Organisation; 2018. Accessed June 13, 2024. https://www.who.int/publications/i/item/WHO-WHE-CPI-2018.26 |
| 42 | *JEE* | *Joint External Evaluation of the International Health Regulations (2005) Core Capacities of Thailand - Mission Report: 31 October-4 November 2022.* World Health Organisation; 2023. Accessed June 13, 2024. https://www.who.int/publications/i/item/9789240080270 |
| 43 | *JEE* | *Joint External Evaluation of IHR Core Capacities of United Arab Emirates.* World Health Organisation; 2017. Accessed June 13, 2024. https://www.who.int/publications/i/item/WHO-WHE-CPI-REP-2017.57 |
| 44 | Scoping review | Handfield R, Apte A, Finkenstadt DJ. Developing supply chain immunity for future pandemic disruptions. *J Humanit Logist Supply Chain Manag*. 2022;12(4):482-501. doi:10.1108/JHLSCM-09-2021-0096 |
| 45 | *JEE* | *Joint External Evaluation of IHR Core Capacities of Australia*. World Health Organisation; 2018. Accessed November 3, 2024. https://www.who.int/publications/i/item/WHO-WHE-CPI-REP-2018.8 |
| 46 | *JEE* | *Joint External Evaluation of IHR Core Capacities of the State of Eritrea.* World Health Organisation; 2017. Accessed June 13, 2024. https://www.who.int/publications/i/item/WHO-WHE-CPI-2017.12 |
| 47 | *JEE* | *Joint External Evaluation of IHR Core Capacities of the Federal Democratic Republic of Ethiopia*. World Health Organisation; 2017. Accessed June 13, 2024. https://www.who.int/publications/i/item/WHO-HSE-GCR-2016.24 |
| 48 | *JEE* | *Joint External Evaluation of IHR Core Capacities of Japan.* World Health Organisation; 2018. Accessed June 13, 2024. https://www.who.int/publications/i/item/WHO-WHE-CPI-REP-2018.23 |
| 49 | *JEE* | *Joint External Evaluation of IHR Core Capacities of the Republic of Liberia.* World Health Organisation; 2017. Accessed June 13, 2024. https://www.who.int/publications/i/item/WHO-WHE-CPI-2017.23 |
| 50 | *JEE* | *Joint External Evaluation of IHR Core Capacities of the Republic of Malawi.* World Health Organisation; 2019. Accessed June 13, 2024. https://www.who.int/publications/i/item/WHO-WHE-CPI-2019.58 |
| 51 | *JEE* | *Joint External Evaluation of IHR Core Capacities of Nepal.* World Health Organisation; 2023. Accessed June 13, 2024. https://www.who.int/publications/i/item/9789240070523 |
| 52 | *JEE* | *Joint External Evaluation of IHR Core Capacities of the Republic of South Africa.* World Health Organisation; 2018. Accessed June 13, 2024. https://www.who.int/publications/i/item/WHO-WHE-CPI-REP-2018.1 |
| 53 | *JEE* | *Joint External Evaluation of IHR Core Capacities of the Republic of Zimbabwe.* World Health Organisation; 2018. Accessed June 13, 2024. https://www.who.int/publications/i/item/WHO-WHE-CPI-REP-2018.24 |
| 54 | *JEE* | *Évaluation externe conjointe des principales capacités du Règlement sanitaire international (2005) de la Guinée*. World Health Organisation; 2024. Accessed September 2, 2024. https://www.who.int/fr/publications-detail/9789240079816 |
| 55 | *JEE* | *Évaluation externe conjointe des principales capacités RSI de la République de Madagascar*. World Health Organisation; 2018. Accessed September 2, 2024. https://www.who.int/fr/publications-detail/WHO-WHE-CPI-REP-2017.66 |
| 56 | *JEE* | *Évaluation externe conjointe des principales capacités RSI de la République du Mali*. World Health Organisation; 2017. Accessed September 2, 2024. https://www.who.int/fr/publications-detail/WHO-WHE-CPI-REP-2017.58 |
| 57 | *JEE* | *Évaluation externe conjointe des principales capacités RSI de la République islamique de Mauritanie*. World Health Organisation; 2017. Accessed September 2, 2024. https://www.who.int/fr/publications-detail/WHO-WHE-CPI-2017.28 |
| 58 | *JEE* | *Joint External Evaluation of the International Health Regulations (‎2005)‎ Core Capacities of Armenia - Mission Report, 4-8 December 2023*. World Health Organisation; 2024. Accessed June 13, 2024. https://www.who.int/publications/i/item/9789240093843 |
| 59 | *JEE* | *Joint External Evaluation of IHR Core Capacities of Georgia.* World Health Organisation; 2019. Accessed June 13, 2024. https://www.who.int/publications/i/item/WHO-WHE-CPI-2019.13 |
| 60 | *JEE* | *Joint External Evaluation of IHR Core Capacities of Kyrgyzstan.* World Health Organisation; 2024. Accessed June 13, 2024. https://www.who.int/publications/i/item/9789240090286 |
| 61 | *JEE* | *Joint External Evaluation of IHR Core Capacities of the Republic of Latvia.* World Health Organisation; 2017. Accessed June 13, 2024. https://www.who.int/publications/i/item/WHO-WHE-CPI-2017.27 |
| 62 | *JEE* | *Joint External Evaluation of IHR Core Capacities of New Zealand.* World Health Organisation; 2019. Accessed June 13, 2024. https://www.who.int/publications/i/item/WHO-WHE-CPI-2019.63 |
| 63 | *JEE* | *Joint External Evaluation of IHR Core Capacities Republic of North Macedonia.* World Health Organisation; 2017. Accessed June 13, 2024. https://www.who.int/publications/i/item/WHO-WHE-CPI-2019.59 |
| 64 | *JEE* | *Joint External Evaluation of IHR Core Capacities of the Republic of Uganda.* World Health Organisation; 2017. Accessed June 13, 2024. https://www.who.int/publications/i/item/WHO-WHE-CPI-REP-2017.49 |
| 65 | *JEE* | *Joint External Evaluation of IHR Core Capacities of Uzbekistan.* World Health Organisation; 2023. Accessed June 13, 2024. https://www.who.int/publications/i/item/9789240070165 |
| 66 | *JEE* | *Évaluation externe conjointe des principales capacités RSI en République Algérienne Démocratique et Populaire*. World Health Organisation; 2023. Accessed September 2, 2024. https://www.who.int/fr/publications-detail/9789240062023 |
| 67 | *JEE* | *Joint External Evaluation of IHR Core Capacities of Cabo Verde*. World Health Organisation; 2020. Accessed September 2, 2024. https://www.who.int/publications/i/item/9789240014435 |
| 68 | *JEE* | *Joint External Evaluation of IHR Core Capacities of the Kingdom of Eswatini.* World Health Organisation; 2018. Accessed June 13, 2024. https://www.who.int/publications/i/item/WHO-WHE-CPI-2018.27 |
| 69 | *JEE* | *Joint External Evaluation of IHR Core Capacities of the State of Kuwait.* World Health Organisation; 2017. Accessed June 13, 2024. https://www.who.int/publications/i/item/WHO-WHE-CPI-REP-2018.11 |
| 70 | Scoping review | Rebmann T, McPhee K, Haas GA, et al. Findings from an Assessment and Inventory of a Regional, Decentralized Stockpile. *Health Secur*. 2018;16(2):119-126. doi:10.1089/hs.2017.0080 |
| 71 | *JEE* | *Joint External Evaluation of IHR Core Capacities of the Republic of Kenya*. World Health Organisation; 2017. Accessed June 13, 2024. https://www.who.int/publications/i/item/WHO-WHE-CPI-REP-2017.44 |
| 72 | *JEE* | *Joint External Evaluation of IHR Core Capacities of the Republic of the Gambia.* World Health Organisation; 2017. Accessed June 13, 2024. https://www.who.int/publications/i/item/WHO-WHE-CPI-REP-2017.61 |
| 73 | Scoping review | Handfield RB, Patrucco AS, Wu Z, Yukins C, Slaughter T. A new acquisition model for the next disaster: Overcoming disaster federalism issues through effective utilization of the Strategic National Stockpile. *Public Adm Rev*. 2024;84(1):65-85. doi:10.1111/puar.13656 |
| 74 | Scoping review | Handfield R, Finkenstadt DJ, Schneller ES, Godfrey AB, Guinto P. A Commons for a Supply Chain in the Post-COVID-19 Era: The Case for a Reformed Strategic National Stockpile. *Milbank Q*. 2020;98(4):1058-1090. doi:10.1111/1468-0009.12485 |
| 75 | Scoping review | Greenawald, L., Moore, S., Yorio, P.L. *Inhalation and Exhalation Resistance and Filtration Performance of Stockpiled Air-Purifying Respirators: Overall Performance of Nearly 4,000 Respirators Sampled from Ten Stockpile Facilities*. Department of Health and Human Services, Centers for Disease Control and Prevention, National Institute for Occupational Safety and Health, NPPTL; 2020. Accessed January 2, 2025. https://www.cdc.gov/niosh/npptl/ppecase/PPE-CASE-P2020-0111.html |
| 76 | Scoping review | Greenawald LA, Moore SM, Wizner K, Yorio PL. Developing a methodology to collect empirical data that informs policy and practices for stockpiling personal protective equipment. *Am J Infect Control*. 2021;49(2):166-173. doi:10.1016/j.ajic.2020.07.010 |
| 77 | *JEE* | *Joint External Evaluation of IHR Core Capacities of the Republic of Indonesia.* World Health Organisation; 2017. Accessed June 13, 2024. https://www.who.int/publications/i/item/WHO-WHE-CPI-REP-2018.9 |
| 78 | *JEE* | *Joint External Evaluation of IHR Core Capacities Republic of the Philippines.* World Health Organisation; 2019. Accessed June 13, 2024. https://www.who.int/publications/i/item/WHO-WHE-CPI-2019.57 |
| 79 | *JEE* | *Joint External Evaluation of IHR Core Capacities of the United Republic of Tanzania.* World Health Organisation; 2017. Accessed June 13, 2024. https://www.who.int/publications/i/item/WHO-HSE-GCR-2016.25 |
| 80 | *JEE* | *Joint External Evaluation of IHR Core Capacities of the United States of America.* World Health Organisation; 2017. Accessed June 13, 2024. https://www.who.int/publications/i/item/WHO-WHE-CPI-2017.13 |
| 81 | *JEE* | *Évaluation externe conjointe des principales capacités RSI de la République de Djibouti*. World Health Organisation; 2018. Accessed September 2, 2024. https://www.who.int/fr/publications-detail/WHO-WHE-CPI-REP-2018.29 |
| 82 | *JEE* | *Évaluation externe conjointe des principales capacités du Règlement sanitaire international (2005) du Togo*. World Health Organisation; 2018. Accessed September 2, 2024. https://www.who.int/fr/publications-detail/WHO-WHE-CPI-REP-2018.31 |
| 83 | *NAPHS* | *National Action Plan for Health Security Federal Republic of Nigeria*. World Health Organisation; 2018. Accessed June 14, 2024. https://extranet.who.int/sph/sites/default/files/document-library/document/Nigeria%20National%20Action%20Plan%20for%20Health%20Security.pdf |
| 84 | *NAPHS* | *The Republic of the Union of Myanmar National Action Plan for Health Security (NAPHS)*. World Health Organisation; 2018. Accessed June 14, 2024. https://extranet.who.int/sph/sites/default/files/document-library/document/National%20Action%20Plan%20for%20Health%20Security%20%28NAPHS%29%202018-2022.pdf |
| 85 | *NAPHS* | *Australian National Action Plan for Health Security*. World Health Organisation; 2019. Accessed June 14, 2024. https://extranet.who.int/sph/sites/default/files/document-library/document/Australia%27s%20National%20Action%20Plan%20for%20Health%20Security%202019-2023.pdf |
| 86 | *JEE* | *Joint External Evaluation of IHR Core Capacities of the Lebanese Republic.* World Health Organisation; 2017. Accessed June 13, 2024. https://www.who.int/publications/i/item/WHO-WHE-CPI-2017.2 |
| 87 | *JEE* | *Joint External Evaluation of IHR Core Capacities of Viet Nam.* World Health Organisation; 2017. Accessed June 13, 2024. https://www.who.int/publications/i/item/WHO-WHE-CPI-2017-21 |
| 88 | *NAPHS* | *Government of Sierra Leone National Action Plan for Health Security (NAPHS)*. World Health Organisation; 2018. Accessed June 14, 2024. https://extranet.who.int/sph/sites/default/files/document-library/document/Sierra%20Leone_%20NAPHS%202018-2022%20Final.pdf |
| 89 | *NAPHS* | *The Republic of Uganda National Action Plan for Health Security*. World Health Organisation; 2019. https://extranet.who.int/sph/sites/default/files/document-library/document/final%20narrative%2011_09_19.pdf |
| 90 | *JEE* | *Joint External Evaluation of IHR Core Capacities of Brunei Darussalam.* World Health Organisation; 2020. Accessed June 13, 2024. https://www.who.int/publications/i/item/9789240006973 |
| 91 | *JEE* | *Joint External Evaluation of IHR Core Capacities of the Republic of Côte d’ivoire.* World Health Organisation; 2017. Accessed June 13, 2024. https://www.who.int/publications/i/item/WHO-WHE-CPI-2017.20 |
| 92 | *NAPHS* | *STATE OF ERITREA MINISTRY OF HEALTH NATIONAL ACTION PLAN FOR HEALTH SECURITY (NAPHS), 2017-2021*. World Health Organisation; 2017. Accessed June 14, 2024. https://extranet.who.int/sph/sites/default/files/document-library/document/Final%20NAPHS%20Eritrea%20Document%2018%20August%202017.pdf |
| 93 | *NAPHS* | *Republic of Liberia Joint National Action Plan for Health Security (NAPHS)*. World Health Organisation; 2018. Accessed June 14, 2024. https://extranet.who.int/sph/sites/default/files/document-library/document/20062018%20Liberia%20NAPHS%20Final%20version.pdf |
| 94 | *JEE* | *Joint External Evaluation of IHR Core Capacities of the Republic of Cameroon*. World Health Organisation; 2017. Accessed September 2, 2024. https://www.who.int/publications/i/item/WHO-WHE-CPI-REP-2017.60 |
| 95 | *JEE* | *Joint External Evaluation of IHR Core Capacities of the Republic of Mauritius.* World Health Organisation; 2019. Accessed June 13, 2024. https://www.who.int/publications/i/item/WHO-WHE-CPI-2019.55 |
| 96 | *JEE* | *Joint External Evaluation of IHR Core Capacities of Senegal.* World Health Organisation; 2018. Accessed June 13, 2024. https://www.who.int/publications/i/item/WHO-WHE-CPI-REP-2017.31 |
| 97 | *JEE* | *Joint External Evaluation of IHR Core Capacities of the Republic of the Sudan.* World Health Organisation; 2017. Accessed June 13, 2024. https://www.who.int/publications/i/item/WHO-WHE-CPI-2017.15 |
| 98 | *JEE* | *Joint External Evaluation of IHR Core Capacities of the Republic of Iraq.* World Health Organisation; 2019. Accessed June 13, 2024. https://www.who.int/publications/i/item/WHO-WHE-CPI-2019.61 |
| 99 | *NAPHS* | *NATIONAL ACTION PLAN FOR HEALTH SECURITY FOR THE REPUBLIC OF NORTH MACEDONIA 2024-2030*. World Health Organisation; 2024. Accessed September 17, 2024. https://cdn.who.int/media/docs/default-source/health-security-preparedness/cap/naphs/naphs-reports/north-macedonia-publication-naphs_web_eng_final.pdf?sfvrsn=9df8cff3_3 |
| 100 | *JEE* | *Joint External Evaluation of IHR Core Capacities of the People’s Republic of Bangladesh.* World Health Organisation; 2017. Accessed June 13, 2024. https://www.who.int/publications/i/item/WHO-HSE-GCR-2016.23 |
| 101 | *NAPHS* | *National Action Plan for Health Security Indonesia*. World Health Organisation; 2020. Accessed June 14, 2024. https://extranet.who.int/sph/sites/default/files/document-library/document/INDONESIA%20NAPHS.PDF |
| 102 | *JEE* | *Joint External Evaluation of IHR Core Capacities in the Republic of Maldives.* World Health Organisation; 2018. Accessed June 13, 2024. https://www.who.int/publications/i/item/WHO-WHE-CPI-2017.30 |
| 103 | *JEE* | *Joint External Evaluation of IHR Core Capacities of the Republic of Tunisia.* World Health Organisation; 2027. Accessed June 13, 2024. https://www.who.int/publications/i/item/WHO-WHE-CPI-REP-2017.45 |
| 104 | *NAPHS* | *National Action Plan for Health Security Ministry of Health Democratic Republic of Timor-Leste*. World Health Organisation; 2020. Accessed June 14, 2024. https://extranet.who.int/sph/sites/default/files/document-library/document/NAPHS_eng_final_with-signature.pdf |
| 105 | *JEE* | *Joint External Evaluation of IHR Core Capacities of Burkina Faso*. World Health Organisation; 2018. Accessed September 2, 2024. https://www.who.int/fr/publications/i/item/WHO-WHE-CPI-REP-2018.12 |
| 106 | *JEE* | Joint External Evaluation of IHR Core Capacities of the Islamic Republic of Afghanistan. World Health Organisation; 2017. Accessed June 13, 2024. https://www.who.int/publications/i/item/WHO-WHE-CPI-REP-2017.43 |
| 107 | *JEE* | Joint External Evaluation of IHR Core Capacities of the Republic of Albania. World Health Organisation; 2017. Accessed June 13, 2024. https://www.who.int/publications/i/item/WHO-WHE-CPI-2017.18 |
| 108 | *JEE* | Joint external evaluation of IHR core capacities of Angola. World Health Organisation; 2021. Accessed June 13, 2024. https://www.who.int/publications/i/item/9789240018266 |
| 109 | *JEE* | Joint External Evaluation of IHR Core Capacities of Azerbaijan. World Health Organisation; 2024. Accessed June 13, 2024. https://www.who.int/publications/i/item/9789240082434 |
| 110 | *JEE* | Joint External Evaluation of IHR Core Capacities of the Kingdom of Bahrain. World Health Organisation; 2017. Accessed June 13, 2024. https://www.who.int/publications/i/item/WHO-WHE-CPI-2017.4 |
| 111 | *JEE* | Joint External Evaluation Of IHR Core Capacities Of The Republic Of Benin. World Health Organisation; 2017. Accessed June 13, 2024. https://www.who.int/publications/i/item/WHO-WHE-CPI-REP-2017.48 |
| 112 | *JEE* | Joint External Evaluation of IHR Core Capacities of the Kingdom of Bhutan. World Health Organisation; 2018. Accessed June 13, 2024. https://www.who.int/publications/i/item/WHO-WHE-CPI-REP-2018.7 |
| 113 | *JEE* | Joint External Evaluation of IHR Core Capacities of the Republic of Botswana. World Health Organisation; 2018. Accessed June 13, 2024. https://www.who.int/publications/i/item/WHO-WHE-CPI-REP-2018.18 |
| 114 | *JEE* | Évaluation Externe Conjointe Des Principales Capacités RSI de La République Du Burundi. World Health Organisation; 2018. Accessed June 13, 2024. https://www.who.int/fr/publications-detail/WHO-WHE-CPI-REP-2018.19 |
| 115 | *JEE* | Joint External Evaluation of IHR Core Capacities of the Kingdom of Cambodia. World Health Organisation; 2017. Accessed June 13, 2024. https://www.who.int/publications/i/item/WHO-WHE-CPI-2017.11 |
| 116 | *JEE* | Évaluation Externe Conjointe Des Principales Capacités RSI de La République Centrafricaine. World Health Organisation; 2019. Accessed June 13, 2024. https://www.who.int/fr/publications/i/item/WHO-WHE-CPI-2019.30 |
| 117 | *JEE* | Joint External Evaluation of IHR Core Capacities of the Republic of Chad. World Health Organisation; 2018. Accessed June 13, 2024. https://www.who.int/publications/i/item/WHO-WHE-CPI-REP-2018.3 |
| 118 | *JEE* | Évaluation Externe Conjointe Des Principales Capacités RSI de l’Union Des Comores. World Health Organisation; 2018. Accessed June 13, 2024. https://www.who.int/fr/publications/i/item/WHO-WHE-CPI-REP-2018.6 |
| 119 | *JEE* | Joint External Evaluation of IHR Core Capacities of the Republic of Congo. World Health Organisation; 2019. Accessed June 13, 2024. https://www.who.int/publications/i/item/WHO-WHE-CPI-2019.60 |
| 120 | *JEE* | Évaluation Externe Conjointe Des Principales Capacités RSI En République Gabonaise. World Health Organisation; 2019. Accessed June 13, 2024. https://www.who.int/fr/publications-detail/WHO-WHE-CPI-2019.18 |
| 121 | *JEE* | Avaliação Externa Conjunta Das Principais Capacidades Do RSI Da Guiné-Bissau. World Health Organisation; 2020. Accessed June 13, 2024. https://www.who.int/pt/publications/i/item/WHO-WHE-CPI-2019.17 |
| 122 | *JEE* | Joint External Evaluation of IHR Core Capacities of the Hashemite Kingdom of Jordan. World Health Organisation; 2017. Accessed June 13, 2024. https://www.who.int/publications/i/item/WHO-WHE-CPI-2017.01 |
| 123 | *JEE* | Joint External Evaluation of IHR Core Capacities of the Kingdom of Lesotho. World Health Organisation; 2017. Accessed June 13, 2024. https://www.who.int/publications/i/item/WHO-WHE-CPI-REP-2017.47 |
| 124 | *JEE* | Joint External Evaluation of IHR Core Capacities of Libya. World Health Organisation; 2019. Accessed June 13, 2024. https://www.who.int/publications/i/item/WHO-WHE-CPI-2019.37 |
| 125 | *JEE* | Joint External Evaluation of IHR Core Capacities of Montenegro. World Health Organisation; 2021. Accessed June 13, 2024. https://www.who.int/publications/i/item/9789240018228 |
| 126 | *JEE* | Joint External Evaluation of IHR Core Capacities of the Republic of Mozambique. World Health Organisation; 2017. Accessed June 13, 2024. https://www.who.int/publications/i/item/WHO-WHE-CPI-2017.19 |
| 127 | *JEE* | Joint External Evaluation of IHR Core Capacities of the Republic of the Union Myanmar. World Health Organisation; 2018. Accessed June 13, 2024. https://www.who.int/publications/i/item/WHO-WHE-CPI-REP-2018.5 |
| 128 | *JEE* | Joint External Evaluation of IHR Core Capacities of Republic of Namibia. World Health Organisation; 2017. Accessed June 13, 2024. https://www.who.int/publications/i/item/WHO-WHE-CPI-REP-2017.36 |
| 129 | *JEE* | Évaluation Externe Conjointe Des Principales Capacités RSI de La République Du Niger. World Health Organisation; 2018. Accessed June 13, 2024. https://www.who.int/fr/publications-detail/WHO-WHE-CPI-2018.33 |
| 130 | *JEE* | Joint External Evaluation of IHR Core Capacities of the Democratic Republic of Sao Tome and Principe. World Health Organisation; 2019. Accessed June 13, 2024. https://www.who.int/publications/i/item/WHO-WHE-CPI-2019.10 |
| 131 | *JEE* | Joint External Evaluation of IHR Core Capacities of the Kingdom of Saudi Arabia. World Health Organisation; 2017. Accessed June 13, 2024. https://www.who.int/publications/i/item/WHO-WHE-CPI-2017.25 |
| 132 | *JEE* | Joint External Evaluation of IHR Core Capacities of Sierra Leone. World Health Organisation; 2023. Accessed June 13, 2024. https://www.who.int/publications/i/item/9789240081376 |
| 133 | *JEE* | Joint External Evaluation of IHR Core Capacities of the Republic of Slovenia. World Health Organisation; 2017. Accessed June 13, 2024. https://www.who.int/publications/i/item/WHO-WHE-CPI-REP-2017.32 |
| 134 | *JEE* | Joint External Evaluation of IHR Core Capacities of the Republic of Somalia. World Health Organisation; 2017. Accessed June 13, 2024. https://www.who.int/publications/i/item/WHO-WHE-CPI-2017.17 |
| 135 | *JEE* | Joint External Evaluation of IHR Core Capacities of the Republic of South Sudan. World Health Organisation; 2018. Accessed June 13, 2024. https://www.who.int/publications/i/item/WHO-WHE-CPI-REP-2018.4 |
| 136 | *JEE* | Joint External Evaluation of IHR Core Capacities of Sri Lanka. World Health Organisation; 2017. Accessed June 13, 2024. https://www.who.int/publications/i/item/WHO-WHE-CPI-REP-2017.33 |
| 137 | *JEE* | Joint External Evaluation of IHR Core Capacities of the Democratic Republic of Timor-Leste. World Health Organisation; 2019. Accessed June 13, 2024. https://www.who.int/publications/i/item/WHO-WHE-CPI-2019.56 |
| 138 | *JEE* | Joint External Evaluation of IHR Core Capacities of Zambia. World Health Organisation; 2017. Accessed June 13, 2024. https://www.who.int/publications/i/item/WHO-WHE-CPI-REP-2017.50 |
| 139 | *JEE* | Joint External Evaluation of IHR Core Capacities of the United Republic of Tanzania - Zanzibar. World Health Organisation; 2017. Accessed June 13, 2024. https://www.who.int/publications/i/item/WHO-WHE-CPI-REP-2017.39 |
| 140 | *NAPHS* | National Action Plan for Health Security (2019-2023): Islamic Republic of Afghanistan. World Health Organisation; 2019. Accessed June 14, 2024. https://extranet.who.int/sph/sites/default/files/document-library/document/NAPHS%20afghanistan%202019.pdf |
| 141 | *NAPHS* | PLAN D’ACTION NATIONAL DE LA SECURITE SANITAIRE DU BENIN 2019 - 2021. World Health Organisation; 2019. Accessed September 17, 2024. https://extranet.who.int/sph/sites/default/files/document-library/document/PANSS%20BENIN%20VF%20OUIDAH%20210319_SIGNE.pdf |
| 142 | *NAPHS* | Republic of South Sudan National Action Plan for Health Security (2020-2024). World Health Organisation; 2020. Accessed June 14, 2024. https://www.afro.who.int/sites/default/files/2020-12/South%20Sudan%20Signed%20NAPHS%20Proposal.pdf |
| 143 | *NAPHS* | National Action Plan for Health Security of Sri Lanka 2019 - 2023. World Health Organisation; 2018. Accessed June 14, 2024. https://extranet.who.int/sph/sites/default/files/document-library/document/NAPHS%20SRI%20LANKA%20-printed%20version.pdf |
| 144 | *NAPHS* | NATIONAL ACTION PLAN FOR HEALTH SECURITY 2017-2021: United Republic of Tanzania. World Health Organisation; 2017. Accessed June 14, 2024. https://extranet.who.int/sph/sites/default/files/document-library/document/NAP_Final_12092017.pdf |
| 145 | *NAPHS* | United States Health Security National Action Plan. World Health Organisation; 2018. Accessed June 14, 2024. https://extranet.who.int/sph/sites/default/files/document-library/document/jee-nap-508.pdf |

**Supplementary material V**

**Table S2.** Characteristics of papers included in a scoping review of seven electronic databases (N=20), including articles published 01 January 2015 to 05 November 2024.

| **Characteristic** | **N (%)** |
| --- | --- |
| Publication Year: |  |
| 2024 | 1 (5) |
| 2023 | 2 (10) |
| 2021 | 4 (20) |
| 2020 | 10 (50) |
| 2018 | 1 (5) |
| 2017 | 1 (5) |
| 2016 | 1 (5) |
| Stockpile*: |  |
| National | 19 (95) |
| Regional | 5 (25) |
| State | 4 (20) |
| County | 3 (15) |
| Hospital | 3 (15) |
| Design*: |  |
| Audit or Site Inspection | 6 (30) |
| Expert Discussion or Workshop | 3 (15) |
| Expert Survey or Interviews | 5 (25) |
| Literature Review | 4 (20) |
| Commentary or Expert Opinion | 6 (30) |

*Some studies used multiple methods and examined multiple stockpile types (therefore total is >100%).
